# Analysis of exhaled nitric oxide and its influencing factors in patients with chronic obstructive pulmonary disease

**DOI:** 10.3389/fmed.2025.1611947

**Published:** 2025-07-07

**Authors:** Ya Shen, Li-Li Yang, Guo-Lan Ning, Xiao-Bao Teng, Jing-Feng Shi, Shun-Shun Cui, Zi-Xiao Cao, Yan-Bei Zhang, Ming-Feng Han

**Affiliations:** ^1^Department of Respiratory and Critical Care Medicine, Fuyang Infectious Disease Clinical College of Anhui Medical University, Fuyang, Anhui, China; ^2^Department of Respiratory and Critical Care Medicine, Fuyang People’s Hospital, Fuyang, Anhui, China; ^3^Geriatric Respiratory and Critical Care, Anhui Geriatric Institute, The First Affiliated Hospital of Anhui Medical University, Hefei, Anhui, China

**Keywords:** exhaled nitric oxide, acute exacerbation, chronic obstructive pulmonary disease, factors, inhaled corticosteroid

## Abstract

**Objectives:**

To compare exhaled nitric oxide (eNO) levels between patients with chronic obstructive pulmonary disease (COPD) and healthy controls, and to investigate factors influencing eNO measurements.

**Methods:**

The study included 115 patients with acute exacerbation of chronic obstructive pulmonary disease (AECOPD), 89 patients with stable COPD, and 70 healthy medical checkups, and the basic data and eNO of the three groups were collected.

**Results:**

Concentration of alveolar Nitric Oxide (CaNO) was higher in the AECOPD group than in the COPD and healthy control groups, nitric oxide concentration in exhaled breath at a flow rate of 200 ml/s (FeNO_200_) was higher in the AECOPD group than in the healthy control group, and the difference was significant. In the AECOPD group, non-smokers and ex-smokers had higher nitric oxide concentration in exhaled breath at a flow rate of 50 ml/s (FeNO_50_) and joint analysis of washout nitric oxide (JawNO) than current smokers. In the healthy control group, FeNO_50_ was higher in non-smokers and ex-smokers than in current-smokers, and JawNO was higher in non-smokers than in current-smokers. In the AECOPD group, non-smokers also had higher FeNO_200_ than current smokers, there was no difference in the comparison of CaNO for different smoking states in the three groups. In the COPD group, BMI was negatively correlated with FeNO_50_, FeNO_200_, and CaNO; height was positively correlated with FeNO_200_ and CaNO. Patients who inhaled Corticosteroids (ICS) had lower FeNO_50_, FeNO_200_, and JawNO than patients who did not inhale ICS in the AECOPD and COPD groups, with a significant difference in comparison, while there was no difference in CaNO. Multiple regression analysis showed that smoking and ICS were the main factors affecting FeNO_50_, FeNO_200_, and JawNO of COPD patients.

**Conclusion:**

The CaNO levels in patients with AECOPD were significantly elevated compared to those with stable COPD and healthy controls. Smoking and the use of ICS were identified as key influencing factors for both FeNO_50_, FeNO_200_, and JawNO. Preliminary observations suggest that BMI and height might exert potential influences on eNO levels in COPD patients, although further investigations are required to confirm these relationships.

## 1 Introduction

Many factors, including environmental ones like smoking, gas pollution, and biofuels, as well as genetic ones like congenital antitrypsin deficiency and gene mutations, can contribute to chronic obstructive pulmonary disease. The disease is also linked to aging and the development of pulmonary function (1). Patients with COPD have pathological changes in their pulmonary vascular system, lung parenchyma, and airways, including structural and inflammatory changes documented (2). Inflammatory changes are characterized by the infiltration of neutrophils (Neu), lymphocytes (Lym), eosinophils (Eos), macrophages, and other inflammatory cells into the lungs. Airway inflammation can be classified into neutrophilic and eosinophilic types (3), with varying treatment choices and disease profiles for each type of inflammation. Studies have shown that although the majority of airway inflammation in COPD patients is of the neutrophilic type, up to 40% of the same patients exhibit eosinophilic type 2 inflammation mediated by cytokines such as interleukin-4, interleukin-5, and interleukin-13 (4).

In recent years, A growing body of studies has demonstrated that Eos can guide the application of corticosteroids and predict future acute exacerbations (5, 6). However, in clinical practice, the Eos cannot correctly reflect patients’ genuine status because some COPD patients take their medication irregularly and utilize corticosteroids. Exhaled nitric oxide (eNO) is a valuable non-invasive tool for assessing airway inflammation (7). It has been suggested by the American Thoracic Society (ATS) and the European Respiratory Society (ERS) as a biomarker of airway inflammation in several respiratory conditions, including asthma and COPD. FeNO_50_ primarily reflects inflammation in the large airways. It is widely used in asthma, while its application in COPD is currently focused on patients with type 2 inflammation, including those with an asthma phenotype and those diagnosed with asthma-COPD overlap syndrome. CaNO and FeNO_200_ mainly reflect inflammation in the peripheral airways and alveoli, thus potentially serving as good indicators for assessing small airway inflammation in COPD patients.

All COPD patients experience acute exacerbations of varied severity and frequency as the illness progresses; these episodes are referred to as AECOPD. Frequent acute exacerbations can worsen the financial burden on families and hasten the deterioration of pulmonary function (8). According to the latest data, COPD ranks as the fourth leading cause of death worldwide, resulting in 3.5 million deaths in 2021, accounting for approximately 5% of global mortality (9). Research has demonstrated that patients with COPD had higher FeNO_50_, FeNO_200_, and CaNO levels than people with simple emphysema, chronic bronchitis, smokers, and healthy populations (10). Additionally, patients with AECOPD have higher FeNO_50_ values than patients with stable-phase COPD (11). No statistically significant disparities were observed in CaNO levels when comparing patients experiencing acute exacerbations of COPD to those with stable COPD, according to a study by Lazar et al. (12) Nevertheless, CaNO levels were found to be elevated in patients with AECOPD and those in the stable phase of COPD, as compared to levels observed in healthy individuals. FeNO_50_ primarily reflects eosinophilic inflammation in the large airways and cannot reflect the inflammation in the small airways and alveoli. Potential influencing factors for eNO levels include smoking status, gender, age, and medication use (13, 14). In contrast, FeNO_200_ and CaNO primarily reflect the inflammation in the small airways and alveoli, which is more applicable in COPD patients. This is due to the fact that small airways serve as the primary site of inflammation in COPD patients. Given the limited research in this area, our study aims to gain deeper insights into eNO levels and their associated influencing factors in COPD patients. We anticipate that these findings will serve as a valuable reference for clinical practice.

## 2 Data and methods

### 2.1 Study population

#### 2.1.1 AECOPD group

One hundred and fifteen patients with AECOPD hospitalized in the Department of Respiratory and Critical Care Medicine, Fuyang Infectious Disease Clinical College of Anhui Medical University, from January 2023 to September 2023, were included. Inclusion criteria: COPD definition based on the Global Initiative for Chronic Obstructive Pulmonary Disease criteria, 2023 edition (15), the ratio of forced expiratory volume in 1 s (FEV_1_) to forced lung capacity (FVC) after inhalation of bronchodilators was < 0.70. AECOPD definition: events characterized by worsening of dyspnea and/or cough and sputum within 14 days in patients with COPD, which may be accompanied by shortness of breath and/or tachycardia, are usually associated with an exacerbation of local or systemic inflammatory responses to respiratory tract infections, air pollution, or other causes. Exclusion criteria: (1) Patients with respiratory diseases such as pulmonary infection, cough variant asthma, bronchodilatation, bronchial asthma, lung malignancy, tuberculosis, etc. (2) Patients with severe cardiovascular and cerebral vascular diseases, hepatic and renal insufficiency, and malignant tumors, etc. (3) Patients with prolonged oral or intravenous glucocorticosteroids (over 3 months). (4) Those who are unable to cooperate with the eNO test and the pulmonary function test. (5) History of acute exacerbation within 4 weeks before hospital admission.

#### 2.1.2 COPD group

A total of 89 patients with stable COPD were enrolled in the study. These individuals attended our hospital’s outpatient clinic at the Department of Respiratory and Critical Care Medicine during the specified period. Inclusion criteria: patients who met the above COPD diagnostic criteria. Exclusion criteria: patients with a history of acute exacerbation within the last 3 months, and the rest were the same as items (1), (2), (3), (4) in the AECOPD group.

#### 2.1.3 Healthy control group

Seventy cases of health checkups in our hospital during the same period were included. Inclusion criteria: age ≥ 40 years, male-dominated, and those with normal ventilatory function in pulmonary function tests. Exclusion criteria: same as (1), (2), (3), (4) in the AECOPD group.

### 2.2 Observational indices and methods

#### 2.2.1 General data

General clinical data of all selected patients were collected, including age, gender, height, weight, smoking history, and body mass index (BMI), BMI was calculated as follows: BMI = weight (kg)/height (m)^2^, inhaled medication of AECOPD and COPD patients were collected. Smokers are defined as > 10 pack-years (PY), non-smokers as patients who stopped greater than 1 year.

#### 2.2.2 Detection of eNO

The eNO test was performed on the day of admission to the hospital in patients with AECOPD (before treatment intervention), at the time of the outpatient visit in patients with COPD, and at the time of the physical examination in those who had a physical exam for health checkups. Following the Official ATS Clinical Practice Guideline and ATS/ERS Recommendations for exhaled breath marker detection (16, 17), the Wuxi Sunvou company’s Nakulan breath analyzer (model: Sunvou-P100) was used. The detection of eNO was performed by an attending physician in our department. Quality control: the analyzer was calibrated daily using certified NO gas standards, and ambient NO levels were confirmed to be < 5 ppb. Expiratory flow and pressure were monitored in real-time to ensure adherence to ATS/ERS criteria. Preparation: subjects avoided nitrate-rich foods, alcohol, and caffeine for ≥ 3 h, and smoking for ≥ 12 h. No exercise or spirometry was performed within 1 h before testing.

##### 2.2.2.1 Test procedure

Let the person sit on a stool to maintain an upright sitting position, use the nose clip to hold the nose, keeping the exhalation filter, first let the person as far as possible to exhale the gas from the lungs completely, and then in a short period, deep inhalation, inhalation is completed after the filter mouthpiece is completely covered lips, according to the system prompted to exhale the gas in the lungs at a flow rate of 50 ml/s (45–55 mL/s range) through a disposable mouthpiece with a bacterial filter, maintaining / closure (oral pressure: 5–20 cm H_2_O). Three valid exhalations (≥ 4 s) were recorded; results were reported as the median value if variability was ≤ 10% (or ≤ 1 ppb for FeNO < 30 ppb). After a short break, the lungs are exhaled in the same manner at a rate of 200 ml/s. JawNO and CaNO calculations were based on dual streams: FeNO = CaNOdual + JawNO/VE + f. VE is the expiratory flow rate (ml/s), and f is a correction factor determined by comparison with the literature on multistream CaNO (18). A limitation of this model is that it assumes constant NO diffusion, ignores axial back diffusion, and may underestimate JawNO during severe blockages.

#### 2.2.3 Detection of pulmonary function

Following the 2019 ATS/ERS technical standards (19), a German Jäger spirometer was applied for the determination, which was done by a specialized technician in our department. Patient instructions: avoid smoking ≥ 1 h, alcohol ≥ 4 h, and vigorous exercise ≥ 30 mins prior. Withhold bronchodilators per protocol: SABA (≥ 4 h), LABA (≥ 12 h).

##### 2.2.3.1 Equipment calibration

Daily volumetric syringe checks (± 3.5% accuracy) and ambient temperature/pressure corrections applied.

##### 2.2.3.2 Test procedure

Let the person sit on a stool in an upright position, using a nasal clip to hold the nose, having an expiratory filter, and wrapping the lips completely around the mouthpiece, the person completes the respiratory maneuvers according to the instructions of the examining technician, and repeats the above test steps twice after the test process has been completed to ensure satisfaction, and takes the best result out of the three times. Further bronchodilatation tests should be performed if the patient has obstructive ventilation dysfunction. The bronchodilatation test requires the subject to inhale 200–400 μg of β2 agonist (e.g., salbutamol) by using a metered-dose inhaler (MDI), and repeat the measurement of FEV_1_ 15–30 min after the inhalation. The specific measurement process is the same as the above. Exhalation filters are specialized and should be retested if there is breath-holding, breath-exchange, leakage, or slobbering during the test.

### 2.3 Statistical analysis

Statistical analysis was performed using SPSS 27.0 and GraphPad Prism 5.0. For categorical variables expressed as frequencies and percentages, the chi-square test was used to compare differences between groups; for continuous variables, those that conformed to normal distribution were expressed as mean ± standard deviation, and *t*-tests or analyses of variance were used to compare between groups. Those that did not meet normal distribution were expressed as median (interquartile spacing, 25%–75%), and Mann-Whitney U or Kruskal-Wallis was used for between-group comparisons. Comparisons between multiple groups were performed using the Dunnett T3 test. Correlation analysis between two variables that conformed to normal distribution was done by Pearson correlation analysis, and those that did not conform to normal distribution were analyzed by Spearman correlation analysis. Multiple linear regression analysis was used to study the factors affecting eNO in patients in the AECOPD and COPD groups. eNO values that did not conform to normal distribution were log-transformed, and dichotomous independent variables must be converted to dummy variables. All *P*-values were tested for two-sidedness, with a test level of α = 0.05 and a statistical difference of *P* < 0.05.

## 3 Results

### 3.1 Comparison of the general data of the three groups

Comparison of general data such as gender, age, height, weight, BMI, and smoking history in the AECOPD group, COPD group patients, and healthy control group showed no statistical difference (*P* > 0.05), there was no difference in the comparison of inhaled corticosteroid (ICS) status between patients in the AECOPD and COPD groups, as depicted in Table 1.

**TABLE 1:** Comparison of general information of patients in the three groups.

Variable	AECOPD group (*n* = 115)	COPD group (*n* = 89)	Healthy control group (*n* = 70)	F/Z/χ^2^ value	*P*-value
Gender (male/ female)	101/14	78/11	58/12	1.068	0.568
Age (year)	70.00 (61.00–74.00)	70.00 (61.00–75.00)	66.50 (57.75–74.00)	3.645	0.162
Height (cm)	166.00 (161.00–170.00)	168.00 (165.00–171.00)	168.00 (162.00–171.00)	3.225	0.199
Weight (kg)	63.22 ± 11.64	64.10 ± 6.12	65.73 ± 8.24	168.089	0.210
BMI (kg/m^2^)	22.95 ± 3.73	22.80 ± 2.10	23.04 ± 2.88	1.352	0.260
Smoking history (yes/no)	95/20	74/15	55/15	0.647	0.724
ICS (yes/no)	67/48	50/39	–	0.089	0.766

### 3.2 Comparison of the eNO of three groups

**FIGURE 1:**
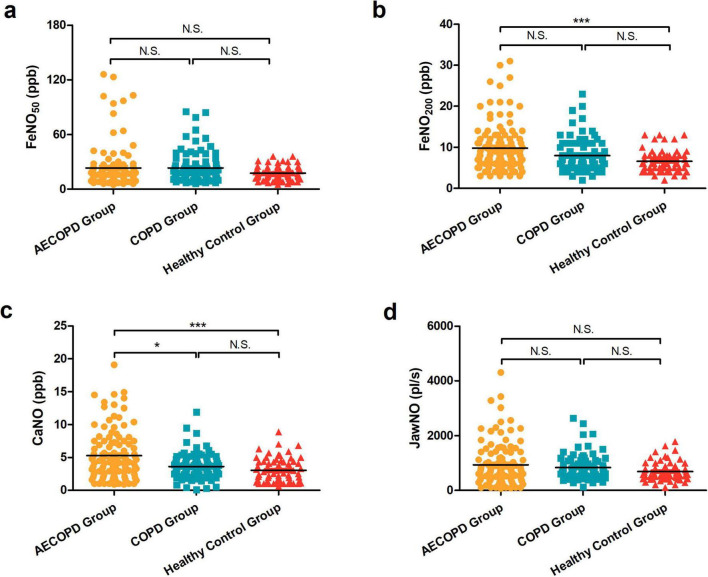
**(a)** Comparison of FeNO_50_ in the acute exacerbation of chronic obstructive pulmonary disease (AECOPD), chronic obstructive pulmonary disease (COPD), and healthy control groups. **(b)** Comparison of FeNO_200_ levels among the three groups. **(c)** Comparison of concentration of alveolar Nitric Oxide (CaNO) levels among the three groups. **(d)** Comparison of joint analysis of washout nitric oxide (JawNO) levels among the three groups. *P < 0.05, ***P < 0.001, N.S, not significant.

As illustrated in Figure 1c, comparative analysis of CaNO revealed significant intergroup variation (*P* < 0.001) across the three cohorts. The AECOPD group exhibited elevated CaNO levels [median (IQR): 4.5 (2.6–7.2) ppb], demonstrating statistically significant differences compared with the COPD group [3.4 (2.5–4.5) ppb, *P* = 0.014] and healthy controls [2.0 (1.4–4.3) ppb, *P* < 0.001]. Notably, no marked disparity was observed between stable COPD patients and healthy subjects (*P* = 0.215). These findings imply pronounced eosinophilic inflammation in distal airways and alveolar regions during COPD exacerbations, potentially associated with heightened airway hyperresponsiveness and mucus hypersecretion in acute phases.

Regarding fractional exhaled nitric oxide at 200 mL/s (FeNO_200_), measurements for AECOPD group [8.0 (6.0–12.0) ppb], COPD group [7.0 (5.0–10.0) ppb], and healthy control group [6.0 (5.0–8.0) ppb] showed differential patterns (Figure 1b). While significant elevation persisted in the AECOPD group versus the healthy control group (*P* < 0.001), neither the AECOPD group vs. the COPD group (*P* = 0.092) nor the COPD group vs. the healthy control group (*P* = 0.098) achieved statistical significance. This suggests preferential involvement of small airway eosinophilic inflammation during acute exacerbations.

In contrast, evaluation of conventional expiratory flow FeNO_50_ measurements [16.0 (11.0–23.0) ppb in AECOPD group; 17.0 (12.5–27.0) ppb in COPD group; 16.0 (12.0–22.3) ppb in healthy control group] failed to reach statistical significance (*P* = 0.334, Figure 1a). JawNO in the three groups were [706.0 (408.0–1206.0) pl/s, 723.0 (522.0–983.0) pl/s, 617.0 (462.0–804.3) pl/s], respectively, with no statistically significant difference in the three group comparisons (*P* = 0.214, Figure 1d). This observation underscores the limited discriminative capacity of standard-flow NO analysis in distinguishing COPD clinical states from healthy physiology.

### 3.3 Comparison of eNO in three groups with different genders

**FIGURE 2:**
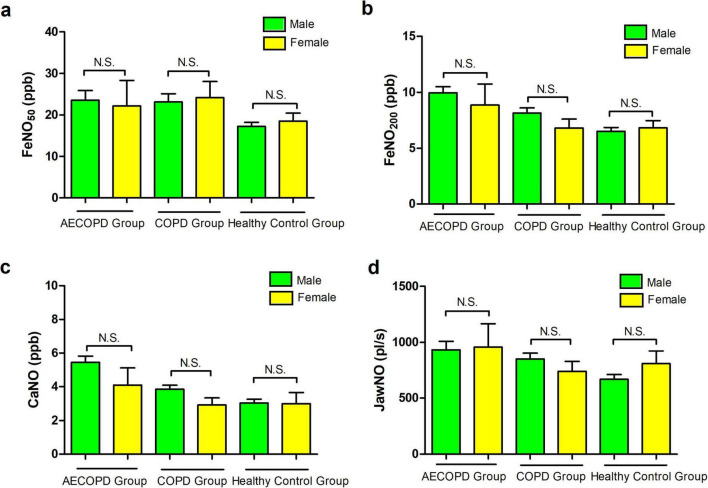
**(a)** Comparison of FeNO_50_ levels between genders in the acute exacerbation of chronic obstructive pulmonary disease (AECOPD) group, chronic obstructive pulmonary disease (COPD) group patients, and healthy control population. **(b)** Comparison of FeNO_200_ levels between different genders in the three groups. **(c)** Comparison of concentration of alveolar Nitric Oxide (CaNO) levels between different genders in the three groups. **(d)** Comparison of joint analysis of washout nitric oxide (JawNO) levels between different genders in the three groups. N.S, not significant.

The differences in FeNO_50_, FeNO_200_, CaNO, and JawNO in the AECOPD group, COPD group patients, and healthy control group were not statistically significant when compared with different genders (*P* > 0.05), as illustrated in Figures 2a–d.

### 3.4 Comparison of eNO in three groups with different smoking statuses

**FIGURE 3:**
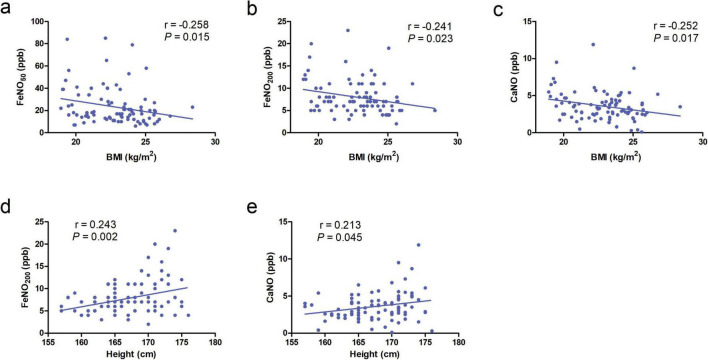
**(a–c)** Correlation of body mass index (BMI) with FeNO_50_, FeNO_200_, and concentration of alveolar Nitric Oxide (CaNO) in the chronic obstructive pulmonary disease (COPD) group. **(d,e)** Correlation of height with FeNO_200_ and CaNO in the COPD group.

Comparative analysis of eNO across smoking status revealed distinct biological patterns (Figure 3). In the AECOPD group, FeNO_50_ demonstrated significant intergroup variation among non-smokers, current smokers, and ex-smokers (*P* < 0.001). *Post hoc* comparisons confirmed elevated FeNO_50_ in both non-smokers and ex-smokers versus current smokers (*P* < 0.001, *P* = 0.002), without significant difference between cessation subgroups (*P* = 1.000). Parallel observations in the COPD group showed 70% higher FeNO_50_ in non-smokers than current smokers (*P* = 0.011), though ex-smokers showed intermediate values without statistical significance. Healthy control group mirrored this trend with non-smokers and ex-smokers exhibiting 40%–50% higher FeNO_50_ than current smokers, demonstrating complete post-cessation recovery. These findings establish FeNO_50_ as a sensitive marker for smoking-modulated eosinophilic inflammation, particularly noting its 68% rebound during acute exacerbations in quitters, informing anti-inflammatory therapy strategies.

At a 200 mL/s flow rate, non-smokers in the AECOPD group maintained elevated FeNO_200_ compared to current smokers (*P* = 0.020), while the COPD group and healthy control groups showed no smoking-related differences (*P* > 0.05), suggesting FeNO_200_’s unique utility in detecting acute-phase small airway inflammation. Crucially, CaNO remained stable across all cohorts, with no statistically detectable smoking effects (*P* > 0.05), confirming CaNO’s reliability for peripheral eosinophilic inflammation assessment independent of tobacco exposure variables.

Similar to FeNO_50_, JawNO also differed across smoking status in the three groups; JawNO was lower in current smokers than in non-smokers in the AECOPD group and the COPD group, and JawNO was greater in quitters than in current smokers (*P* < 0.05). In the healthy population, JawNO was lower in current smokers than in non-smokers (*P* = 0.028). The above results are shown in Table 2.

**TABLE 2:** Comparison of exhaled nitric oxide (eNO) levels in the three groups with different smoking statuses.

Smoking status	FeNO_50_ (ppb)	FeNO_200_ (ppb)	CaNO (ppb)	JawNO (pl/s)
**AECOPD group**				
Non-smokers	18.5 (12.0–46.5)	8.0 (6.0–20.8)	4.8 (2.0–9.3)	855.5 (588.5–2268.0)
Current smokers	11.0 (8.0–15.5)*	7.0 (5.0–8.0)*	4.2 (2.9–5.0)	545.0 (287.0–767.5)*
Ex-smokers	18.5 (14.0–24.3)^#^	9.0 (6.8–13.0)	4.8 (2.4–7.2)	763.0 (448.0–1411.0)^#^
**COPD group**				
Non-smokers	23.0 (17.0–53.0)	6.0 (5.0–12.0)	4.4 (2.4–6.1)	823.0 (702.0–1284.0)
Current smokers	13.5 (10.3–19.3)*	7.0 (5.0–9.8)	4.8 (2.6–6.7)	592.0 (472.0–753.0)*
Ex-smokers	18.0 (14.0–26.3)	7.0 (6.0–10.3)	4.8 (2.9–6.8)	803.0 (572.0–1084.0)^#^
**Healthy control group**				
Non-smokers	21.0 (17.0–24.0)	7.0 (5.0–8.0)	2.5 (1.0–4.4)	763.0 (502.0–1010.0)
Current smokers	14.0 (9.0–18.0)*	6.0 (4.0–7.0)	3.1 (1.4–4.1)	562.0 (418.5–749.3)*
Ex-smokers	20.0 (15.0–25.0)^#^	7.0 (5.5–9.0)	4.1 (1.6–6.9)	723.0 (504.5–1054.0)

*vs. non-smokers, *P* < 0.05. ^#^vs. current smokers, *P* < 0.05.

### 3.5 Correlation analysis of age, height, weight, BMI, and eNO in three groups

No significant correlations were observed between age, height, weight, or BMI with FeNO_50_, FeNO_200_, CaNO, or JawNO levels in AECOPD patients and healthy controls (all *P* > 0.05). In contrast, the COPD group demonstrated negative correlations between BMI and all three biomarkers: FeNO_50_ (r = −0.258, *P* = 0.015), FeNO_200_ (r = −0.241, *P* = 0.023), and CaNO (r = −0.252, *P* = 0.017) (Figures 3a–c). These findings suggest that obesity-related systemic inflammation may suppress localized airway inflammatory responses, with metabolic disturbances associated with elevated BMI potentially disrupting nitric oxide synthesis pathways. Additionally, height exhibited positive correlations with FeNO_200_ (r = 0.243, *P* = 0.002), CaNO (r = 0.213, *P* = 0.045), likely attributable to increased airway surface area in taller individuals leading to greater cumulative release of inflammatory markers (Figures 3d, e).

### 3.6 Effect of ICS on eNO in the AECOPD group and COPD group

**FIGURE 4:**
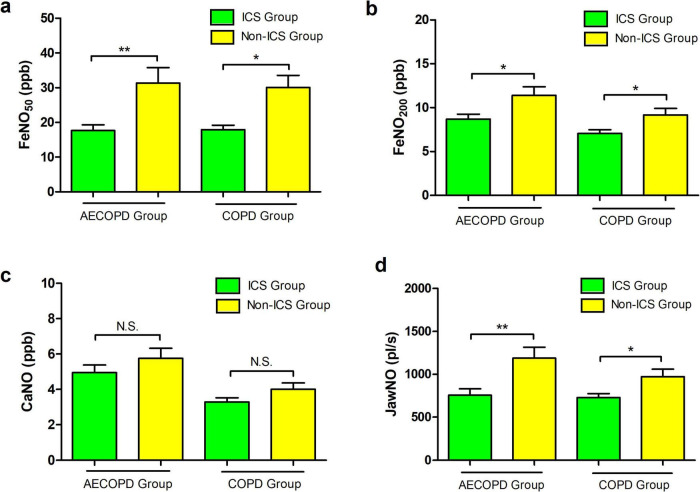
**(a)** Comparison of FeNO_50_ levels between inhaled corticost (ICS) and non-ICS patients in the acute exacerbation of chronic obstructive pulmonary disease (AECOPD) and chronic obstructive pulmonary disease (COPD) groups. **(b)** Comparison of FeNO_200_ levels between ICS and non-ICS patients in the AECOPD and COPD groups. **(c)** Comparison of concentration of alveolar Nitric Oxide (CaNO) levels between ICS and non-ICS patients in the AECOPD and COPD groups. **(d)** Comparison of joint analysis of washout nitric oxide (JawNO) levels between ICS and non-ICS patients in the AECOPD and COPD groups. *P < 0.05, **P < 0.01.

Statistical comparisons between ICS-treated and non-ICS subgroups revealed distinct inflammatory biomarker profiles. In the AECOPD cohort, FeNO_50_ levels measured 16.0 (9.0–21.0) ppb for ICS users versus 20.5 (14.0–31.5) ppb for non-users (*P* = 0.003), with FeNO_200_ levels at 8.0 (5.0–11.0) ppb and 9.5 (7.0–13.8) ppb, respectively (*P* = 0.018), JawNO values were 626.0 (346.0–1002.0) pl/s and 813.5 (571.5–1757.0) pl/s, respectively (*P* = 0.003). No between-group differences emerged for CaNO levels [4.3 (2.4–6.9) ppb vs. 4.7 (2.8–8.2) ppb, *P* = 0.308]. Parallel analysis in stable COPD patients showed similar patterns: FeNO_50_ levels were 16.0 (12.0–20.3) ppb with ICS versus 23.0 (14.0–40.0) ppb without (*P* = 0.01), FeNO_200_ levels were 7.0 (5.0–8.0) ppb versus 8.0 (6.0–12.0) ppb (*P* = 0.03), and JawNO values were 712.5 (502.0–863.0) pl/s and 823.0 (602.0–1204.0), respectively (*P* = 0.042). CaNO measurements again showed non-significant variations [3.0 (2.4–4.5) ppb vs. 3.8 (2.7–4.9) ppb, *P* = 0.149]. These data demonstrate ICS effectively reduces FeNO_50_, FeNO_200_, and JawNO concentrations across disease states while exhibiting limited impact on distal airway/alveolar inflammation reflected by CaNO (Figure 4).

### 3.7 Multiple linear regression analysis of factors influencing eNO in the patients of AECOPD group

The multiple linear regression analysis of factors influencing eNO parameters in AECOPD patients revealed distinct patterns across the measured biomarkers (Table 3). Use of inhaled corticosteroids (ICS) and current smoking status emerged as consistent predictors for FeNO_50_, FeNO_200_, and JawNO levels. Specifically, non-ICS demonstrated significant positive associations with FeNO_50_, FeNO_200_, and JawNO, whereas current smokers exhibited inverse relationships with these parameters. For CaNO, no variables reached statistical thresholds. Demographic and anthropometric factors, including age, gender, and BMI, failed to demonstrate significant associations with any eNO parameters across all models.

**TABLE 3:** Multiple linear regression analysis of factors influencing exhaled nitric oxide (eNO) in the acute exacerbation of chronic obstructive pulmonary disease (AECOPD) group.

Factors	FeNO_50_			FeNO_200_			CaNO			JawNO		
	β	SE	*P*-value	β	SE	*P*-value	β	SE	*P*-value	β	SE	*P*-value
ICS	−0.315	0.051	< 0.001	−0.203	0.042	0.027	−0.076	0.062	0.421	−0.311	0.067	< 0.001
Current smokers (vs. non-smokers and ex-smokers)	−0.299	0.059	0.001	−0.225	0.050	0.018	−0.014	0.073	0.886	−0.256	0.079	0.005
Female gender (vs. male)	−0.077	0.078	0.383	−0.133	0.066	0.155	−0.184	0.096	0.061	−0.013	0.103	0.889
Age (years)	−0.074	0.003	0.409	0.014	0.003	0.882	0.074	0.004	0.447	−0.119	0.004	0.190
BMI (kg/m^2^)	0.021	0.007	0.819	−0.045	0.006	0.632	−0.084	0.009	0.396	0.039	0.009	0.671

### 3.8 Multiple linear regression analysis of factors influencing eNO in the patients of the COPD group

Multiple linear regression analysis of factors influencing eNO parameters in the COPD group revealed distinct associations across biomarkers (Table 4). Using inhaled corticosteroids (ICS) demonstrated consistent negative correlations with FeNO_50_, FeNO_200_, and JawNO, while non-smoking status positively influenced FeNO_50_. BMI approached statistical significance for FeNO_50_, FeNO_200_, and CaNO. No significant relationships were observed for CaNO, with ICS and non-smokers failing to reach statistical thresholds. Demographic variables other than BMI did not show consistent predictive value with all eNO parameters.

**TABLE 4:** Multiple linear regression analysis of factors influencing exhaled nitric (eNO) in the chronic obstructive pulmonary disease (COPD) group.

Factors	FeNO_50_			FeNO_200_			CaNO			JawNO		
	β	SE	*P*-value	β	SE	*P*-value	β	SE	*P*-value	β	SE	*P*-value
ICS	−0.280	0.051	0.005	−0.250	0.039	0.016	−0.181	0.064	0.088	−0.216	0.044	0.035
Non-smokers (vs. current smokers and ex-smokers)	0.237	0.077	0.035	0.110	0.060	0.351	0.024	0.097	0.839	0.222	0.067	0.058
Female gender (vs. male)	−0.054	0.085	0.619	−0.162	0.066	0.156	−0.131	0.107	0.262	−0.158	0.073	0.161
Age (years)	−0.057	0.003	0.579	−0.125	0.002	0.252	−0.068	0.004	0.545	−0.051	0.003	0.631
BMI (kg/m^2^)	−0.283	0.012	0.005	−0.271	0.009	0.010	−0.258	0.015	0.017	−0.203	0.011	0.051

## 4 Discussion

Nitric oxide (NO), a key biomarker of airway inflammation, is produced by nitric oxide synthase (NOS). Pathogen infection induces inducible NOS (iNOS), leading to elevated NO levels (20). NO enhances Th2-mediated inflammation by activating eosinophils and other immune cells (21). Exhaled NO measurement reflects type 2 inflammatory conditions like asthma and allergic rhinitis (22). Advanced techniques now enable segmental airway NO assessment, including nasal NO at a flow rate of 10 mL/s (FnNO), FeNO_50_, FeNO_200_, JawNO, CaNO, etc. An essential feature of COPD is small airway disease. The current assessment of small airway disease mainly includes lung function measurements, such as expiratory flow testing, Impulse Oscillometry (IOS), lung clearance index (LCI) measurements, etc., and quantitative airway indexes by high-resolution CT. In addition, biomarker measurements are a new way to assess small airway disease, such as Club Cell Protein 16 (CC16), Plasminogen Activator Inhibitor-1 (PAI-1), and Matrix Metalloproteinases (MMPs) measurements (23). As a non-invasive and reproducible marker of airway inflammation, eNO is strongly associated with type 2 airway inflammatory state in COPD patients.

Our study subjects were primarily COPD patients, excluding those with comorbid asthma. By comparing the levels of CaNO, FeNO_50_, FeNO_200_, and JawNO in AECOPD patients with those in stable COPD patients and healthy individuals, we further understand the levels of eNO in COPD patients and seek to identify the most clinically valuable biomarkers. After conducting a statistical analysis of the data, we found that the CaNO in patients with AECOPD was higher than that in patients with stable COPD and healthy individuals, and the FeNO_200_ was higher than that in healthy individuals, with significant statistical differences in both comparisons. Although FeNO_50_ and JawNO in both AECOPD and stable COPD patients were higher than those in healthy individuals, there were no statistically significant differences in these comparisons.

The scarcity of research on eNO in COPD is noteworthy. A study that enrolled the same population as our investigation, including patients with AECOPD, patients with stable COPD, and healthy individuals, showed no significant differences in FeNO_50_ levels among the three groups. The results of the study also demonstrated that FeNO_200_ serves as a strong predictor of peripheral airway/alveolar inflammation in COPD patients, with the area under the receiver operating characteristic (ROC) curve reaching 0.841, demonstrating better predictive value compared to CaNO (AUC = 0.707) (9). Another study by Fan et al. (17) showed that FeNO_200_ was significantly higher in patients with stable chronic obstructive pulmonary disease (COPD) and AECOPD than in healthy controls. Brindicci et al. (24) reported that CaNO levels in COPD patients were significantly higher than in both smoking COPD risk groups and non-smoking healthy controls, corroborating our findings. The observed elevation in inflammatory markers in the peripheral small airways, without corresponding increases in central airway inflammation, may be associated with heightened oxidative stress following frequent acute exacerbations in COPD. This pathological state promotes enhanced secretion of cellular inflammatory mediators (25), which subsequently upregulates the activity of both neuronal nitric oxide synthase (nNOS) and inducible nitric oxide synthase (iNOS) in the peripheral airways. Another study provided a foundation for the observed increase in FeNO_200_ and CaNO among COPD patients. By examining the NOS levels in peripheral lung tissues, they identified varying degrees of iNOS and nNOS upregulation, which was found to be correlated with the patient’s lung function classification (26). Among the three biomarkers, CaNO demonstrated consistent elevation across stable COPD and acute exacerbation phases, with more prominent increases observed in AECOPD patients. These fluctuations likely correlate with airway hyperresponsiveness and mucus hypersecretion during exacerbations, suggesting CaNO may hold superior clinical utility in COPD management.

Concurrently, we analyze factors that may affect eNO test results in healthy individuals and patients with COPD during stable and acute exacerbation phases, further to grasp the stability and advantages of each biomarker. The influence of gender on eNO levels remains equivocal, with a preponderance of evidence suggesting that FeNO_50_ is elevated in males compared to females. A study from Dunedin, New Zealand, demonstrated that FeNO_50_ levels were approximately 25% lower in females than in males (27). All participants of this research were 32 years old at the time of the study thus controlling for age-related variations in FeNO_50_. A Korean study, encompassing 166 healthy non-smokers aged 20–68 years, also reported a statistically significant disparity in FeNO_50_ between genders, with males exhibiting higher levels (35.7 ± 13.2 ppb) than females (26.0 ± 14.2 ppb) (28). Olivieri et al. (29), in their analysis of eNO in 204 healthy non-smokers, observed gender-based differences, with males exhibiting higher FeNO_50_, FeNO_100_ (fractional exhaled nitric oxide at 100 mL/s), and FeNO_200_ levels than females. The gender disparity was particularly pronounced for FeNO_100_ and FeNO_200_. Drawing from the existing literature, we hypothesize that CaNO levels are more markedly elevated in male patients with stable COPD and AECOPD. The reasons for these inter-gender discrepancies may include: (1) variations in blood nitrate concentrations, a metabolite of nitric oxide, which could lead to gender-specific differences in endogenous airway NO (30); (2) the inhibitory effect of estrogen on the expression of iNOS, thereby reducing eNO levels (31); (3) genetic polymorphisms in the nNOS gene, which has been linked to lower eNO levels in females, although no correlation was identified between nNOS genotype and eNO levels in males (32). Our findings indicate that there were no significant statistical differences in FeNO_50_, FeNO_200_, CaNO, and JawNO levels among male and female patients across the three groups. The reason for this outcome may be attributed to the disproportionate gender ratio in our study population, with a relatively more minor number of females, and the failure to control for factors such as smoking and age.

The impact of smoking on FeNO_50_ has been extensively documented, with the majority of studies indicating a reduction in FeNO_50_ levels among smokers. However, the influence of smoking on CaNO remains less consistent. Lehtimaki et al. (33) reported that current smokers with COPD exhibited significantly lower FeNO_50_ and CaNO levels compared to non-smokers. A separate study, which included non-smoking healthy individuals, the smoking COPD risk population, and COPD patients, revealed that FeNO_50_, FeNO_100_, and FeNO_200_ levels were lower in the smoking COPD risk population compared to non-smokers and COPD patients. Interestingly, CaNO levels were higher in the smoking COPD risk population than in non-smoking healthy individuals, suggesting a diminished impact of smoking on CaNO (24). Hogman et al. (34) compared FeNO_50_ levels between 20 smokers and 30 non-smokers, noting significantly lower FeNO_50_ levels in smokers. However, nine smokers who abstained from smoking for 4 weeks showed no significant difference in FeNO_50_ levels compared to non-smokers, implying that smoking cessation for over 4 weeks may normalize FeNO_50_ levels. Nerpin et al. (15) corroborated these findings, demonstrating reduced FeNO_50_ in smoking individuals compared to non-smokers.

Our study compared FeNO_50_, FeNO_200_, JawNO, and CaNO among AECOPD patients, COPD patients in a stable phase, and healthy individuals across different smoking statuses. The results indicated that current smokers in all three groups had lower FeNO_50_ levels compared to those who had quit smoking and non-smokers, with statistically significant differences observed in AECOPD patients and healthy individuals, but not in COPD patients, where the difference between current and former smokers was not statistically significant. Similar to FeNO_50_, JawNO was lower in current smokers than in non-smokers in the AECOPD and COPD groups, and JawNO was greater in quitters than in current smokers. In the healthy population, JawNO was lower in current smokers than in non-smokers. Additionally, in the AECOPD group, current smokers had lower FeNO_200_ than non-smokers, which was statistically significant. In contrast, no significant differences in CaNO levels were observed across different smoking statuses in all three groups. These findings suggest that while smoking significantly affects FeNO_50_ and JawNO levels, its influence on CaNO is less pronounced. The underlying mechanisms may involve the high concentration of NO in smoke, which can be toxic to airway epithelial cells, leading to the downregulation or inactivation of NOS activity and, consequently, the inhibition of NO synthesis. Additionally, the high concentration of superoxide in smoke rapidly reacts with NO to form reactive nitrogen species, accelerating NO elimination (35).

The associations between demographic characteristics (age, height, weight, BMI) and biomarkers (FeNO_50_, FeNO_200_, CaNO, JawNO) were assessed in COPD patients and healthy individuals. The results indicated that in patients with stable COPD, BMI was negatively correlated with FeNO_50_, FeNO_200_, and CaNO, while height was positively correlated with FeNO_200_ and CaNO. In contrast, no significant correlations were observed among all these parameters in AECOPD patients and healthy individuals. A pan-Asian study examining FeNO_50_ levels in healthy adolescents aged 5–18 reported a positive correlation with age and an inverse relationship with body weight (36). Nerpin et al. (15) identified a positive correlation between FeNO_50_ and age in adults, with no significant associations observed with height, body weight, or BMI. Hogman et al. (38) noted a more pronounced increase in CaNO levels in middle-aged and older adults, particularly those aged 50 years and above. This trend may be attributed to several factors: (1) the diminished capacity for CaNO diffusion into the pulmonary circulation and subsequent clearance by hemoglobin, which declines linearly with age, resulting in reduced CaNO clearance in older individuals; (2) the decreased efficiency of macrophages in clearing invading pathogens in the elderly, potentially leading to low-grade inflammation in the lower respiratory tract and elevated CaNO levels (37, 38).

Another study reported a positive correlation between adult height and FeNO_50_, with an increase of 10 cm in height corresponding to a rise of 1.11 ppb for males and 0.90 ppb for females in FeNO_50_ (39). Al-Shamkhi et al. (40) found a correlation between body weight and FeNO_50_ (r = 0.10, *P* = 0.02), but no such correlation with BMI, consistent with another study (41). A recent study demonstrated an inverse correlation between FeNO_200_ and height in COPD patients (r = −0.301, *P* < 0.05), whereas FeNO_50_ and CaNO showed no significant associations with height in this cohort. Conversely, asthmatics exhibited positive CaNO-height correlations (r = 0.328, *P* < 0.05), with FeNO_50_ and FeNO_200_ remaining statistically independent of height (40). Given the lack of consensus on the influence of these factors on eNO, future studies with larger cohorts are warranted to elucidate these relationships more definitively.

Inhaled corticosteroid is a cornerstone therapy for asthma and is also recommended for specific subsets of patients with COPD. Specifically, ICS is indicated for the following person: (1) patients with comorbid bronchial asthma or an asthmatic phenotype; (2) patients with blood eosinophil counts (Eos) ≥ 300/μl, or Eos ≥ 100/μl coupled with a history of frequent acute exacerbations, defined as one or more moderate exacerbations leading to hospitalization or two or more moderate exacerbations. A study demonstrated that FeNO_50_ levels in bronchial asthma patients who received ICS 1 week before FeNO_50_ testing were significantly lower compared to those who did not, with quartile values of 16.8 (15.3, 18.5) ppb versus 20.4 (19.0, 21.9) ppb, respectively (42). In a Chinese study involving 214 AECOPD patients, subjects were categorized into elevated and normal groups based on FeNO_50_ at 25 ppb. The findings indicated a significantly higher prevalence of ICS use in the elevated group, suggesting that FeNO_50_ could predict ICS utilization. Receiver Operating Characteristic (ROC) curve analysis revealed a statistically significant distinction between the two groups, characterized by an Area Under the Curve (AUC) of 0.631, with a 95% confidence interval ranging from 0.526 to 0.736, and associated with a *P*-value of 0.022 (12). A meta-analysis by Lim et al. (43) reported a significant reduction in FeNO_50_ levels following ICS treatment in ex-smokers with COPD, whereas no significant improvement was observed in current smokers.

The mechanism by which ICS reduce FeNO_50_ is primarily attributed to their anti-inflammatory effects on airway epithelial cells in asthma and COPD patients. In these patients, inflammatory cells such as T-helper 2 (Th2) cells, mast cells, and eosinophils release IL-4 (interleukin-4)/IL-13 (interleukin-13), which upregulate iNOS expression via the Signal Transducer and Activator of Transcription 6 (STAT-6) pathway. Corticosteroids exert inhibitory effects on various inflammatory and precursor cells, dampening this pathway (44). Our study compared the levels of FeNO_50_, FeNO_200_, JawNO, and CaNO in patients with COPD in the stable phase and during acute exacerbations, with and without inhaled corticosteroids (ICS). The results revealed that patients receiving ICS had significantly lower levels of FeNO_50_, JawNO, and FeNO_200_ compared to those not on ICS, with statistically significant differences observed. However, the two groups had no significant difference in CaNO levels. Thus, ICS therapy effectively reduces FeNO_50_, JawNO, and FeNO_200_ levels in COPD patients, necessitating dynamic dosage adjustments based on disease stages. However, its minimal impact on CaNO likely relates to inefficient drug particle deposition in distal airways, providing theoretical rationale for combining ultrafine-particle inhalers. Current limited evidence requires further studies for validation and mechanistic clarification.

In this study, multivariate linear regression analysis was performed to assess the factors influencing eNO levels in the AECOPD and COPD groups. The results indicated that ICS use was the primary determinant affecting both FeNO_50_, FeNO_200_, and JawNO groups. Smoking status, however, exhibited a differential impact, significantly altering FeNO_50_, FeNO_200_, and JawNO levels in the AECOPD group, while only influencing FeNO_50_ in the COPD group. Furthermore, among physiological parameters, BMI was found to exert a measurable influence on FeNO_50_, FeNO_200_, and CaNO levels specifically in COPD patients. Large-scale studies investigating the determinants of eNO in COPD patients remain scarce to date. A recent Austrian population-based study focusing on healthy individuals revealed a positive correlation between FeNO_50_ and age, with adult females exhibiting lower FeNO_50_ levels than males. Additionally, multiple covariates—including height, blood eosinophil count, residential environment, total Immunoglobulin E (IgE), and the FEV_1_/FVC ratio (among younger participants)—demonstrated significant associations with FeNO_50_. Cardiovascular conditions emerged as a modifying factor exclusively in older cohorts (45). A multinational investigation conducted across Europe and Australia, involving a large healthy population, reported significantly higher FeNO_50_ levels in males than in females. The analysis further identified smoking status, height, and serum IgE as universal determinants of FeNO_50_ variations. An age-dependent elevation in FeNO_50_ was consistently observed across all participants, with this increase occurring at younger ages in women compared to men (14).

## 5 Conclusion

This study comprehensively investigated exhaled nitric oxide (eNO) levels in COPD patients and healthy individuals, performing detailed statistical analyses of influencing factors. Results revealed elevated CaNO levels in AECOPD patients compared to stable COPD patients and controls, with higher FeNO_200_ levels observed in AECOPD patients versus controls. These findings indicate that eNO elevation, particularly FeNO_200_ and CaNO as small-airway inflammation markers, strongly correlates with exacerbated airway inflammation during COPD progression, especially in acute exacerbations, as potential indicators for disease status differentiation.

Smoking significantly decreased FeNO_50_ and JawNO in both COPD patients and healthy subjects. It minimally affected FeNO_200_ and CaNO, suggesting its predominant impact on large-airway inflammation rather than small-airway/alveolar inflammation. This provides critical insights for interpreting smoking-related inflammatory patterns in COPD. ICS emerged as a key modulator of FeNO_50_, FeNO_200_, and JawNO in COPD patients due to its anti-inflammatory effects in large/small airways. However, its limited CaNO reduction reflects suboptimal deposition efficiency in distal airways. Physiological parameters (height, BMI) showed moderate correlations with eNO levels in stable COPD, potentially linked to individual inflammatory response heterogeneity.

This study has certain limitations that should be acknowledged. Firstly, the range of influencing factors analyzed was relatively limited. Additionally, the research did not include repeated eNO measurements to assess reproducibility. Future investigations will address these gaps by refining the experimental design to yield more comprehensive and reliable findings. While offering novel clinical references for airway inflammation assessment, exacerbation prediction, and treatment optimization, the findings necessitate validation through multicenter, large-scale studies to establish eNO’s role as a COPD biomarker fully.

## Data Availability

The raw data supporting the conclusions of this article will be made available by the authors, without undue reservation.
